# Aggressive angiomyxoma of the liver: a case report and literature review

**DOI:** 10.1186/s40792-017-0365-4

**Published:** 2017-08-23

**Authors:** Koki Sato, Masahiro Ohira, Seiichi Shimizu, Shintarou Kuroda, Kentaro Ide, Kohei Ishiyama, Tsuyoshi Kobayashi, Hiroyuki Tahara, Noriyuki Shiroma, Koji Arihiro, Michio Imamura, Kazuaki Chayama, Hideki Ohdan

**Affiliations:** 1 Department of Gastroenterological and Transplant Surgery, Applied Life Sciences, Institute of Biomedical & Health Sciences, Hiroshima University, 1-2-3, Kasumi, Minami-ku, Hiroshima, 734-8551 Japan; 2 Department of Pathology, Graduate School of Biomedical Sciences, Hiroshima University, 1-2-3, Kasumi, Minami-ku, Hiroshima, 734-8551 Japan; 3 Department of Medicine and Molecular Science, Division of Frontier Medical Science, Programs for Biomedical Research, Graduate School of Biomedical Sciences, Hiroshima University, Hiroshima, Japan

**Keywords:** Aggressive angiomyxoma (AAM), Liver, Immunohistochemistry

## Abstract

**Background:**

Aggressive angiomyxoma (AAM) is a rare mesenchymal tumor that occurs almost exclusively in the soft tissue of the pelvis and perineum. AAM has both locally infiltrative and recurrent characteristics. Very few cases of AAM occurring outside of the pelvis and perineum have been reported. Here, we report a case of AAM originating in the liver of a 33-year-old female patient.

**Case presentation:**

A 33-year-old woman underwent S8 subsegmentectomy after clinical diagnosis of a mucinous cystic neoplasm of the liver. Histological analysis revealed a tumor composed of spindle-shaped cells with vascular proliferation in a myxoid stroma. Immunohistochemically, the tumor cells stained positively for CD34, estrogen receptor (ER), and progesterone receptor (PgR) and negatively for S-100, EMA, CK19, CD99, HMB45, and α-smooth muscle actin. The tumor was diagnosed as AAM originating from the liver. The patient received no adjuvant chemotherapy. No sign of recurrence or distant metastasis has been noted for 10 months after the surgery.

**Conclusions:**

We here report a second case of AAM originating from the liver, which is an uncommon location for this particular tumor.

## Background

Aggressive angiomyxoma (AAM) is a rare mesenchymal tumor with myxoid and vascular components that usually occurs in the pelvi-perineal region of females, as first described by Steeper and Rosai in 1983 [1].

AAM lesions nearly exclusively arise in the soft tissues of the pelvi-perineal region of adult women. However, uncommon cases have been reported [2–6] in which the neoplasm has features consistent with AAM but occurs in the head, neck, or lung regions.

Here, we also discuss the differential characteristics of AAM occurring outside of the pelvi-perineal region.

To the best of our knowledge, only one case of AAM originating from the liver has been previously reported, and that case was reported by Qi et al. in 2015 [7]. This report describes an additional case of primary AAM arising from the right lobe of the liver in a 33-year-old woman.

## Case presentation

**Fig. 1:**
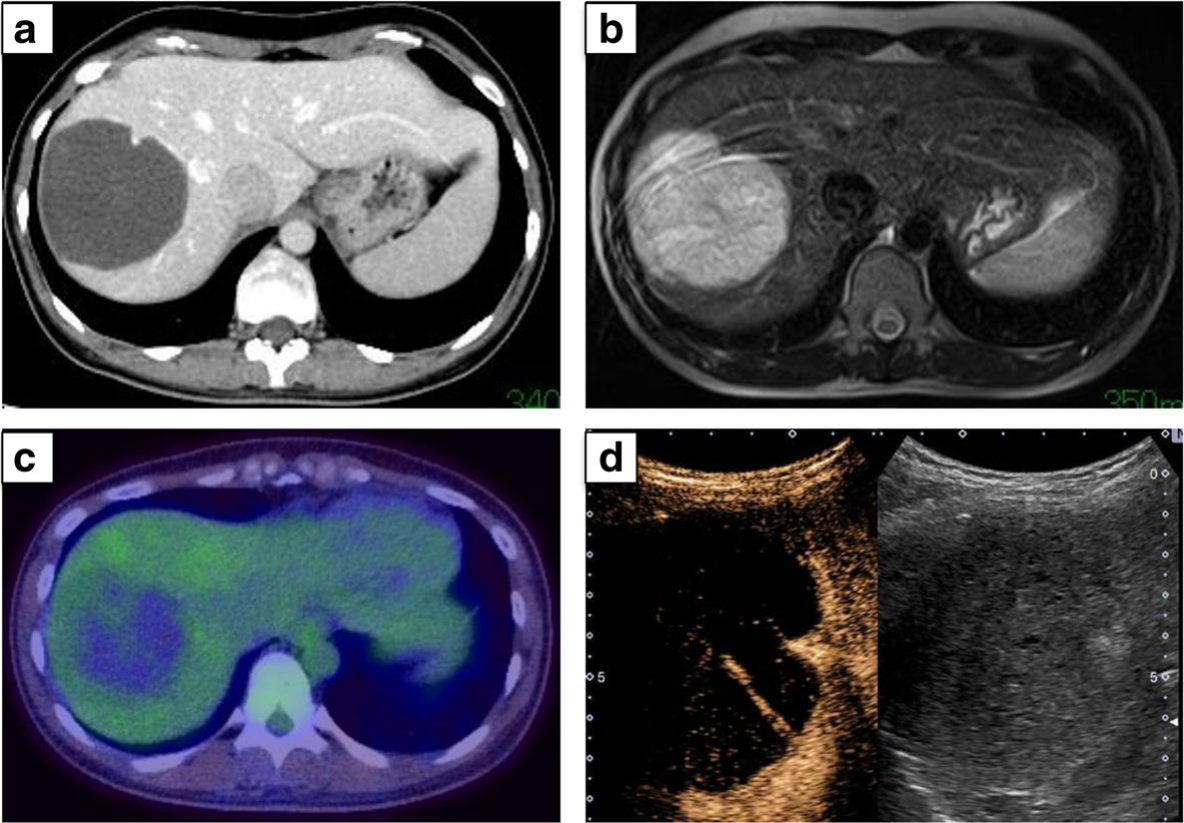
**a** CT scan showing a lobular, cystic tumor with highly viscous liquid. **b** MRI showing hyper-intense regions on T2. **c** FDG-PET showing the lack of uptake in the tumor. **d** Sonazoid-enhanced ultrasound showing a solid, hypovascular tumor with an enhanced internal partition wall

**Fig. 2:**
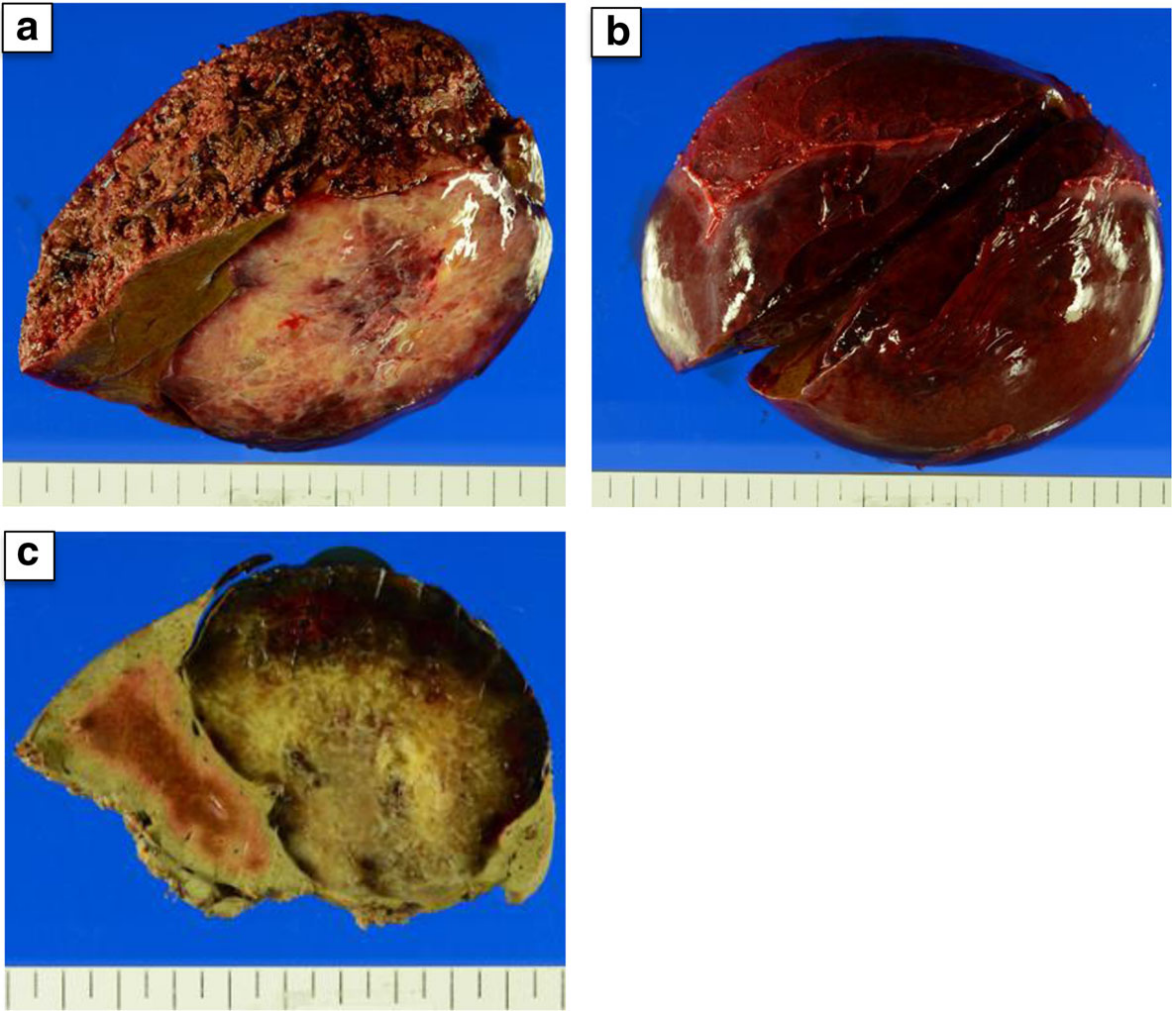
**a** Isolated specimens showing a soft, bulky tumor with a homogenous glistening cut surface. **b**, **c** The cut surface of the tumor showing myxoid and vascular components

**Fig. 3:**
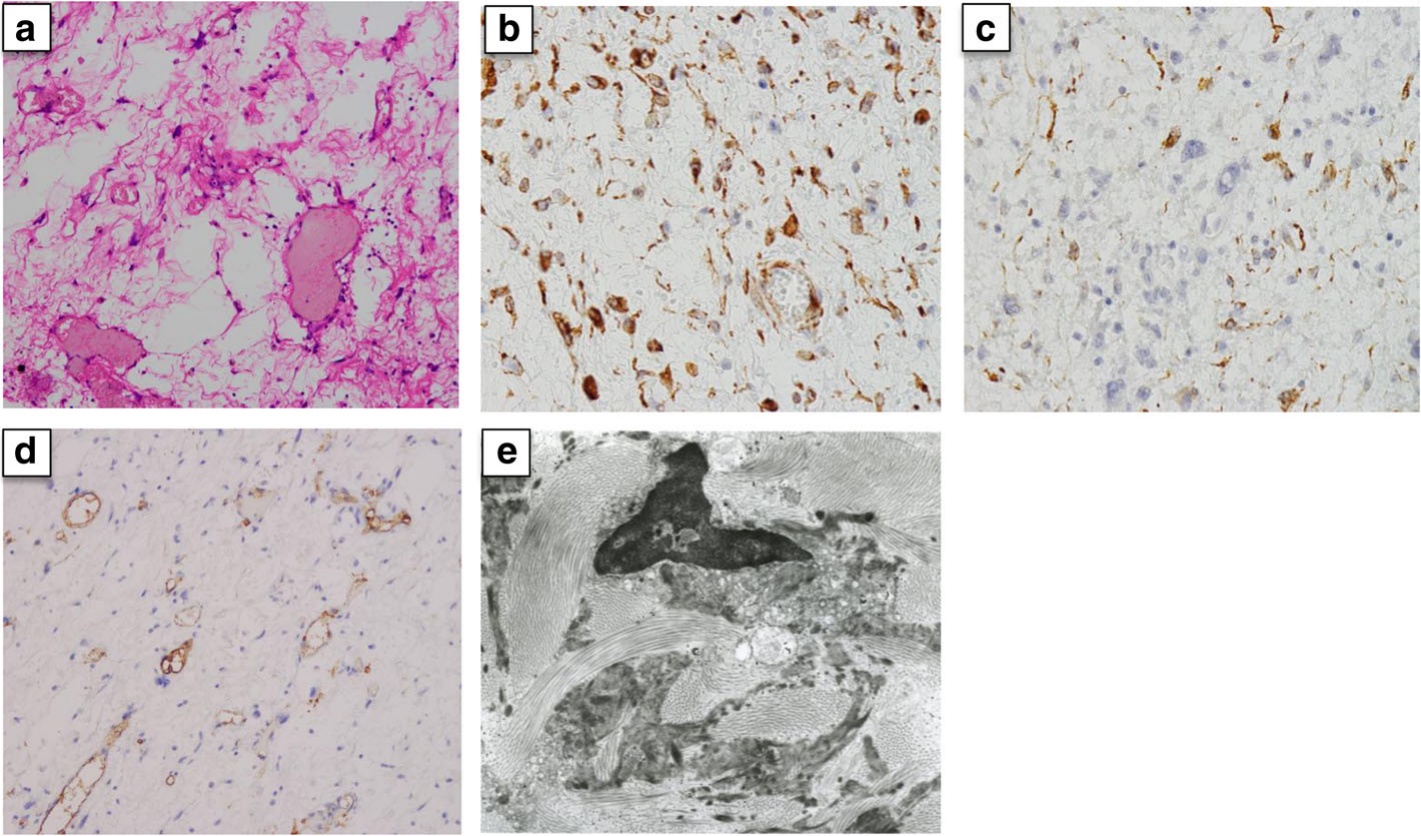
**a** Hematoxylin-eosin staining showing stellate-formed cells with prominent vascularity and myxoid matrix tissue (× 100 magnification). **b** Positive immunoreactivity for vimentin in the tumor (× 400 magnification). **c** Positive immunoreactivity for desmin in the tumor (× 400 magnification). **d** Positive immunoreactivity for CD34 in the tumor (× 100 magnification). **e** Electron microscope image showing collagen fibers extended around the nucleus

A 33-year-old Japanese woman was referred to our hospital for treatment of a hepatic mass, 8 cm in diameter, located in segment 8. The tumor was detected by abdominal ultrasound screening. Contrast computed tomography (CT) showed a lobular, cystic tumor pooling highly viscous liquid (Fig. 1a). With magnetic resonance imaging (MRI), the mass was iso-intense or less commonly hypo-intense (compared to the muscle) on T1 and hyper-intense on T2 (Fig. 1b). Gadolinium-ethoxybenzyl-diethylenetriamine pentaacetic acid (Gd-EOB-DTPA) contrast MRI showed that the tumor’s internal dividing wall was contrasted. The tumor did not show uptake on fluorodeoxyglucose-positron emission tomography (FDG-PET) (Fig. 1c). Ultrasound showed a solid, hypoechoic mass, with a boundary that was slightly unclear and internal inhomogeneity. Sonazoid-enhanced ultrasound showed a solid, hypovascular tumor, with an enhanced internal partition wall (Fig. 1d). Liver function tests were unremarkable. Blood tumor markers, including carcinoembryonic antigen, α-fetoprotein, and carbohydrate antigen 19–9 were within the normal ranges. The patient was not a carrier of hepatitis B or C, and her human immunodeficiency virus serology was nonreactive. From these findings, she was diagnosed with mucinous cystic neoplasm of the liver and underwent S8 subsegmentectomy of the liver. Macroscopic examination showed a tumor (8.0 × 7.5 cm) that was well circumscribed, oval, and rubbery (Fig. 2a). The cut surface of the tumor showed myxoid and vascular components (Fig. 2b, c). Histopathological examination showed that the tumor was composed of spindle-shaped cells with vascular proliferation in a myxoid stroma (Fig. 3a). Immunohistochemically, the tumor cells stained positively for vimentin (Fig. 3b), desmin (Fig. 3c), CD34 (Fig. 3d), ER, and PgR and negatively for S-100, EMA, CK19, CD99, HMB45, and α-smooth muscle actin. Electron microscope image showed that collagen fibers extended around the nucleus (Fig. 3e).

The postoperative course was uneventful, and she was discharged from the hospital 9 days after surgery. The patient was followed for 10 months postoperatively. There were no signs of recurrence or distant metastasis.

## Discussion

AAM is a rare mesenchymal neoplasm occurring mostly in the pelvi-perineal region of adults, as first described by Steeper and Rosai in 1983 [1]. AAM is a rare but well-described tumor; Halder K, et al. reviewed less than 250 cases in their literature [8]. AAM has both locally infiltrative and recurrent characteristics. About 90% of patients are women, usually of reproductive age [9]. A few cases have been described in males, typically in sites around the genital tract [10]. Although most cases of AAM occur in the pelvi-perineal region and genital tract, a few cases have been described in other locations, such as the head, neck, lung, and liver (Table 1). Two of six (33%) patients were preoperatively diagnosed with AAM by biopsy. It is often difficult to diagnose AAM prior to removal because of the rarity of this tumor and lack of characteristic clinical features. In our case, we did not perform liver biopsy because of the preoperative examination diagnosed with mucinous cystic neoplasm of the liver. Thus, most cases are diagnosed on the basis of the histology findings after surgical excision.

**Table 1:** Summary of AAM originating from outside the pelvi-perineal region

Author (reference)	Year	Location	Age	Sex	Size (cm)	Immunohistochemical exam	Surgical treatment	Resection margin (mm)	Adjuvant therapy	Disease-free survival (months)	Recurrence
Magalhaes F.TD [2]	1995	Larynx	63	F	2.0	• Negative: S-100	Tumorectomy	Unknown	No	1	Local recurrence
Yamashita Y [3]	2004	Oral floor	8	F	2.0	• Positive: vimentin • Negative: S-100, CD34	Surgical excision	10	No	24	None
Pai CY [4]	2008	Supraclavicular fossa	48	M	12 × 10	• Positive: vimentin, desmin, muscle-specific actin, ER • Negative: cytokeratin, S100, PgR	Wide excision after preoperative biopsy	Unknown	No	6	None
Choi YD [5]	2008	Lung	70	F	6.0 × 5.5	• Positive: vimentin, desmin, actin, ER, PgR • Negative: cytokeratin, S-100, CD34, bcl-2	Thoracoscopic surgery after preoperative needle biopsy	Unknown	No	8	None
Sylvester DC [6]	2010	Larynx	47	M	4.0 × 2.5	• Negative: S-100, desmin, CD34, SMA	Laryngofissure approach excision biopsy and covering tracheostomy	Unknown	No	48	None
Qi S [7]	2015	Liver	50	F	2.0 × 2.0	• Positive: vimentin, CD34, SMA • Negative: desmin, S-100, Ki-67, EMA, ER, PgR, CD99, CD10, CAM5.2, CK19	Partial hepatectomy	Unknown	No	6	None
Present case	2016	Liver	33	F	8.0 × 7.5	• Positive: vimentin, desmin, CD34, ER, PgR • Negative: S-100, EMA, CD99, HMB45, CK19	Subsegmentectomy	2	No	10	None

*Abbreviations*: *AAM* aggressive angiomyxoma, *ER* estrogen receptor, *F* female, *M* male, *PgR* progesterone receptor

Regarding diagnosis with AAM, the characteristic MRI findings of AAM include the following: On T1-weighted MRI, it has low signal intensity. That is, it is of the same signal intensity as the skeletal muscle. On T2-weighted MRI, it has high signal intensity. These appearances likely relate to the loose myxoid matrix and high water content of angiomyxoma [11]. The CT findings demonstrated that the tumor, later identified as AAM, had a well-defined margin and an attenuation less than that of a muscle [11]. On ultrasonography, AAM appears as a hypoechoic or cystic mass [12]. In our case, the liver mass showed high signal intensity on T2-weighted MRI, as an attenuated mass on CT, and as a hypoechoic cystic region on ultrasound (Fig. 1). In our case, CT and FDG-PET unable to detect any mass in other regions including the pelvi-perineal region. Therefore, the AAM was concluded as of liver origin.

Immunohistochemically, AAM has been found to be positive for vimentin, desmin, CD 34, ER, PgR, and α-smooth muscle actin and negative for MSA and S-100 [13]. Table 1 described immunohistochemical features of AAM which occurred in other than pelvi-perineal region. In our case, immunostaining was positive for vimentin, desmin, CD34, and ER, PgR and was negative for S100, EMA, CD99, HMB45, and CK19, which was conclusive of the diagnosis of AAM. In the liver, myxoid or fibrous tumors with abundant vasculature are not frequent. The differential diagnosis of these tumors includes myxoid neurofibroma, angiomyolipoma (AML), and angiomyofibroblastoma (AMF). Myxoid neurofibroma showed a positive immunohistochemical staining for S-100, and AML showed specifically positive for HMB-45. AMF is also characterized by the immunoreactivities of vimentin, desmin, and CD34. AAM can be distinguished from the aforementioned tumors by the proliferation of spindle- or satellite-shaped cells in a myxoid background with a prominent vascular component [1]. From these findings, our case was distinguished from AMF by the features including subcutaneous location, smaller tumor size, sharply circumscribed margins, and delicate smaller tumor vessels.

The ideal treatment of AAM is complete surgical excision with a wide margin, because AAM is a locally aggressive tumor. However, another study asserted that the recurrence rate in patients with narrow surgical margins is not higher than that of patients with wide surgical margins [14]. Although we aim for complete resection, incomplete or partial resection is acceptable, especially when high operative morbidity is anticipated or preservation of fertility is an issue. Long-term follow-up and careful monitoring with imaging techniques are essential for timely identification of recurrence [15]. Distant metastasis is rarely reported [16, 17].

Radiation therapy and chemotherapy should not be recommended for AAM because the mitotic index of AAM is usually low. Hormonal treatment could be used as an adjuvant therapy for recurrent and residual tumors, because most AAM express estrogen and progesterone receptors [14]. In cases with large tumors requiring extensive resection, arterial embolization and/or hormonal treatment may be used initially, followed by surgical resection [18]. Regarding AAM derived from extra-pelvic regions, all six patients underwent surgical resection without any additional adjuvant therapies (Table 1).

## Conclusions

This report describes the second case of AAM originating from the liver, which is an uncommon location for this particular tumor. Reporting a large series of these tumors may lead to a better understanding of how AAM may occur outside the pelvi-perineal region.

## Abbreviations

CA 19–9: Cancer antigen 19–9
CEA: Carcinoembryonic antigen
CT: Computed tomography
GIST: Gastrointestinal stromal tumor
H&E: Hematoxylin and eosin
MRI: Magnetic resonance imaging
PET: Positron emission tomography

## References

[1] Steeper TA, Rosai J (1983). Aggressive angiomyxoma of the female pelvis and perineum. Report of nine cases of a distinctive type of gynecologic soft-tissue neoplasm. Am J Surg Pathol.

[2] Teixeira-De-Magalhaes F, Pardal-De-Oliveira F (1995). Angiomyxoma of larynx. Report of one case of a myxoid fibrohistiocytic lesion. Pathologica.

[3] Yamashita Y, Tokunaga O, Goto M (2004). Aggressive angiomyxoma of the oral floor: report of a case. J Oral Maxillofac Surg.

[4] Pai CY, Nieh S, Lee JC, Lo CP, Lee HS (2008). Aggressive angiomyxoma of supraclavicular fossa: a case report. Head Neck.

[5] Choi YD, Kim JH, Nam JH, Choi C, Na KJ, Song SY (2008). Aggressive angiomyxoma of the lung. J Clin Pathol.

[6] Sylvester DC, Kortequee S, Moor JW, Woodhead CJ, Maclennan KA (2010). Aggressive angiomyxoma of larynx: case report and literature review. J Laryngol Otol.

[7] Qi S, Li B, Peng J, Wang P, Li W, Chen Y (2015). Aggressive angiomyxoma of the liver: a case report. Int J Clin Exp Med.

[8] Haldar K, Martinek IE, Kehoe S (2010). Aggressive angiomyxoma: a case series and literature review. Eur J Surg Oncol.

[9] Wiser A, Korach J, Gotlieb WH, Fridman E, Apter S, Ben-Baruch G (2006). Importance of accurate preoperative diagnosis in the management of aggressive angiomyxoma: report of three cases and review of the literature. Abdom Imaging.

[10] Kaur A, Makhija PS, Vallikad E, Padmashree V, Indira HS (2000). Multifocal aggressive angiomyxoma: a case report. J Clin Pathol.

[11] Outwater EK, Marchetto BE, Wagner BJ, Siegelman ES (1999). Aggressive angiomyxoma: findings on CT and MR imaging. AJR Am J Roentgenol.

[12] Yaghoobian J, Zinn D, Ramanathan K, Pinck RL, Hilfer J (1987). Ultrasound and computed tomographic findings in aggressive angiomyxoma of the uterine cervix. J Ultrasound Med.

[13] Amezcua CA, Begley SJ, Mata N, Felix JC, Ballard CA (2005). Aggressive angiomyxoma of the female genital tract: a clinicopathologic and immunohistochemical study of 12 cases. Int J Gynecol Cancer.

[14] Dahiya K, Jain S, Duhan N, Nanda S, Kundu P (2011). Aggressive angiomyxoma of vulva and vagina: a series of three cases and review of literature. Arch Gynecol Obstet.

[15] Chan YM, Hon E, Ngai SW, Ng TY, Wong LC (2000). Aggressive angiomyxoma in females: is radical resection the only option?. Acta Obstet Gynecol Scand.

[16] Siassi RM, Papadopoulos T, Matzel KE (1999). Metastasizing aggressive angiomyxoma. N Engl J Med.

[17] Blandamura S, Cruz J, Faure Vergara L, Machado Puerto I, Ninfo V (2003). Aggressive angiomyxoma: a second case of metastasis with patient’s death. Hum Pathol.

[18] Han-Geurts IJ, van Geel AN, van Doorn L, den Bakker M, Eggermont AM, Verhoef C (2006). Aggressive angiomyxoma: multimodality treatments can avoid mutilating surgery. Eur J Surg Oncol.

